# Time-dependent mechanical-electrical coupled behavior in single crystal ZnO nanorods

**DOI:** 10.1038/srep09716

**Published:** 2015-05-18

**Authors:** Yong-Jae Kim, Tae Gwang Yun, In-Chul Choi, Sungwoong Kim, Won Il Park, Seung Min Han, Jae-il Jang

**Affiliations:** 1 Division of Materials Science and Engineering, Hanyang University, Seoul 133-791, Korea; 2 Graduate School of EEWS, Korea Advanced Institute of Science and Technology, Daejeon 305-701, Korea

## Abstract

Nanoscale time-dependent mechanical-electrical coupled behavior of single crystal ZnO nanorods was systematically explored, which is essential for accessing the long-term reliability of the ZnO nanorod-based flexible devices. A series of compression creep tests combined with *in*-*situ* electrical measurement was performed on vertically-grown single crystal ZnO nanorods. Continuous measurement of the current (*I*)-voltage (*V*) curves before, during, after the creep tests revealed that *I* is non-negligibly increased as a result of the time-dependent deformation. Analysis of the *I*-*V* curves based on the thermionic emission-diffusion theory allowed extraction of nanorod resistance, which was shown to decrease as time-dependent deformation. Finally, based on the observations in this study, a simple analytical model for predicting the reduction in nanorod resistance as a function of creep strain that is induced from diffusional mechanisms is proposed, and this model was demonstrated to be in an excellent agreement with the experimental results.

Recent progress in micro- and nanoelectronics has mostly been focused on the development of new-types of electronic and optoelectronic systems, consisting of electric components that are devised on flexible, rollable, and stretchable substrates^1^,^2^,^3^. As candidate materials for the components in the new devices, inorganic one-dimensional nanomaterials (such as nanowires and nanorods) are attracting attention due to their intrinsically superior carrier mobility than that in most organic materials, in addition to their ability to accommodate large strains without failure^4^,^5^,^6^. Such advancement in electronics accompanies the change in operating environments. For example, these new devices can be rolled or bent for storage and transport, which results in stresses imposed on each component of the device. Although the stresses applied to each of the components must be much lower than the critical stresses for yielding or instantaneous failure, additional deformation can still occur if the components are exposed to lower stresses for a relatively long period of time; i.e., the possibility of “time-dependent” permanent deformation, often referred to as creep. Consideration of the creep deformation is especially important for nanomaterials even at low temperatures due to the increased role of the surface enhanced diffusion^7^. In this regard, very recently, the room-temperature nanoscale creep of ZnO nanorods was reported, which revealed that creep can occur even at room temperature in the nanorods under the stresses within elastic regime and the creep deformation is more pronounced for smaller nanorods and higher applied stresses^7^.

Time-dependent deformation may induce microstructural and geometrical changes in the materials and hence may alter the corresponding electrical performance^8^,^9^,^10^. For instance, the resistivity of Al_2_O_3_ single crystal was found to increase with increasing dislocation density upon progress of creep deformation^10^. Such changes in the electrical property can be detrimental to devices, since a small change in defect density can lead to large fluctuation in the device performance, especially when the size of electric components become down to the nanoscale^11^,^12^. Therefore, to guarantee long-term reliability of nanomaterial-based flexible electronics, better understanding of time-dependent mechanical-electrical coupled behavior of nanomaterials is essential. Despite this practical importance, however, almost no attempt has been made to systematically explore the creep-induced change in electrical properties at the nanoscale. A main possible reason for this limitation is the experimental impediment which may come from the absence of suitable equipment and established technique for investigating the time-dependent mechanical-electrical coupled behavior of nanomaterials. Until now, there are only a few studies that report coupled mechanical-electrical behavior, and specimens considered had dimensions in the microns regime^13^,^14^,^15^. In one report, both ends of the microwires were fixed on a polymer substrate and the time-dependent change in electrical behavior was explored while subjecting the substrate to bending strain^13^. Meanwhile, a more quantitative measurement of the mechanical response using the conventional nanomechanical testing machine equipped with electric measurement system was adopted to investigate the coupled behavior of the micro-sized arrays (e.g., carbon nanotube turf)^14^. However, both of these studies used a viscous polymer substrate and/or an adhesion layer that would also contribute to creep strain leading to technical difficulties in separation of the time-dependent coupled behavior from the specimen of interest. Moreover, the time-dependent mechanical-electrical coupled behavior has been rarely investigated in the individual nanomaterials.

With all these in mind, in the present study, we report a systematic investigation of how the room temperature nanoscale creep can affect the electrical response of nanomaterials through a series of *in-situ* electro-mechanical tests. Vertically grown single crystal ZnO nanorods were chosen as a testing system, since ZnO is known to be one of the most promising nanomaterials today due to its excellent performance and multifunctionality with wide applications in flexible devices with electrical, optical, sensing, and energy harvesting fields^2^,^16^,^17^. Especially, ZnO nanomaterials have been reported to show room-temperature creep behavior^7^ and well-organized geometry of bottom-up grown nanorods is proper for nanoscale uniaxial tests, such as pillar compression tests. It is noteworthy that this bottom-up method can eliminate the issues associated with possible surface damage which can be generated during the nanopillar sample preparation by focused ion beam (FIB) milling^18^,^19^. Electrical contact resistance (ECR) characterization was utilized to measure the *in-situ* electrical properties while imposing precise mechanical loading using uniaxial pillar compression methods nanoscale to overcome the difficulties in conventional nanoindentation creep tests such as the complex stress distribution underneath the indenter^20^. The mechanical-electrical coupled behavior during creep of single crystal ZnO nanorods was analyzed to determine the resistance evolution during creep deformation. A model for prediction of nanorod resistance is proposed as a function of creep strain by using the thermionic emission-diffusion theory for the metal-semiconductor junction at the top contact of the ZnO nanorod.

## Results

### Testing condition and electrical behavior

**Figure 1:**
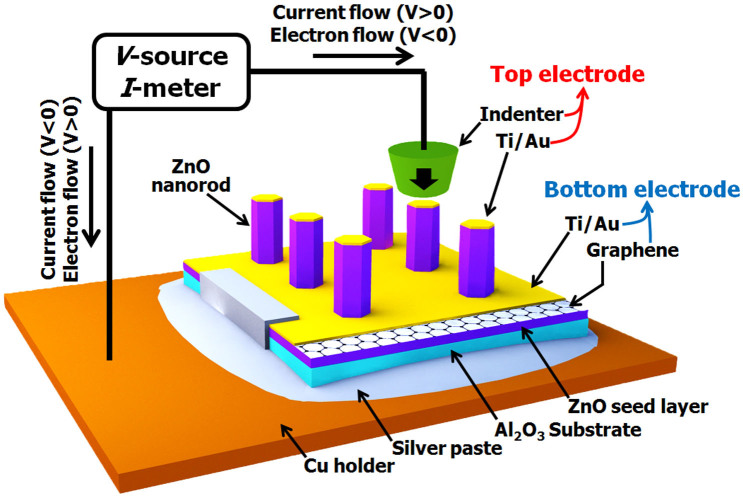
Schematic illustration of testing setup for *in-situ* mechanical-electrical characterization.

**Figure 2:**
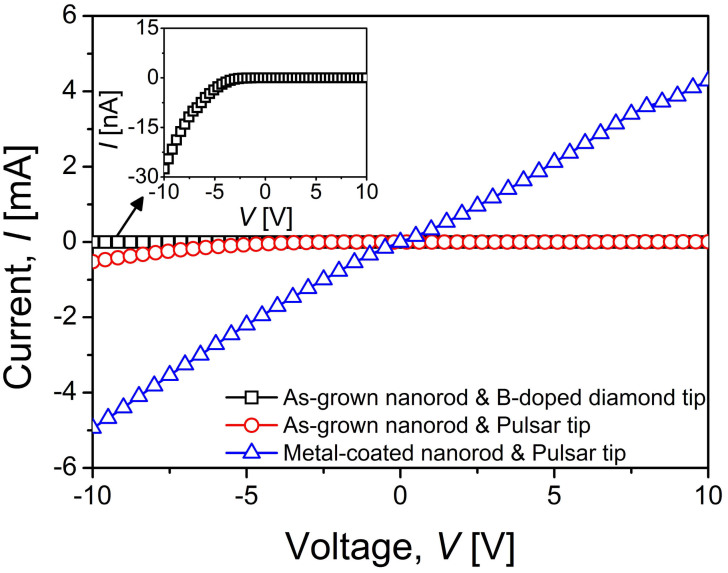
Representative examples of *I*-*V* curves obtained from as-grown and metal-coated ZnO nanorods with flat-ended B-doped diamond tip and Pulsar tip. The *I*-*V* curve from as-grown nanorods with diamond tip is enlarged in the inset image.

Fig. 1 shows a schematic of the testing setup for nanoscale mechanical-electric coupled experiments where the direction of current and electron flow is also indicated (for details, see the methods section). Current-voltage (*I-V*) curves of both the as-grown and the metal-coated ZnO nanorods were obtained with both the B-doped diamond tip and the Pulsar tip as shown in Fig. 2. In the case of as-grown nanorods, Pulsar tip produces sub-mA level *I* values that are much higher than those obtained with B-doped diamond tip (tens of nA scale, as shown in the inset of Fig. 2), suggesting that the use of Pulsar indenter is appropriate for obtaining reliable electrical contact. The B-doped diamond tip was reported to have inhomogeneity in electric conductivity due to nonuniform B doping^21^. Since uniform electric contact is important for obtaining reliable results, especially in nanomaterials due to small contact area, the Pulsar indenter tip, which is electrically more homogeneous than B-doped diamond tip, was adopted for the main experiments introduced in the following sections. Our results indicate that the Pulsar tip produces more reproducible results, while the B-doped tip caused large scatters due to its electric non-uniformity.

The *I*-*V* curves of the as-grown nanorods were analyzed as shown in Fig. 2, which exhibits rectifying characteristics with *I* flowing only at large negative *V*. This non-symmetric nature is typically observed in the semiconductor-based devices due to the formation of Schottky barrier at metal-semiconductor (M-S) junction, which can play a crucial role in the electrical transport and then *I*-*V* relation^22^. To observe the creep-induced change in electrical properties by amplifying the *I-V* relation, additional metal layers were deposited on the nanorods to reduce contact resistance; Ti was first deposited followed by Au to prevent oxidation of the Ti. During the vertical sputter deposition of metal layers, the possibility of the metal deposition on the side walls of ZnO nanorods (having a taper angle of <1°) is expected to be very low due to the highly directional character of the sputtering process. This is supported by the scanning electron microscopy (SEM) observations that the surface of the side walls are very smooth side walls while the top surface are relatively rough due to the deposition. Even if very small amount of metals are deposited on the side walls, their effect may be almost negligible and the possibility of short-circuit between top and bottom of a metal-coated nanorod can be neglected^23^, which is confirmed by the fact that the coated nanorods still exhibit a nonlinear *I*-*V* behavior. In addition, we could not found metal components (i.e., Au and Ti) on the side surface of nanorod through Energy Dispersive X-Ray Spectrometer (EDS) analysis, while the existence of metal on its top surface was confirmed (for the results of EDS, see Supplementary Information). Finally, the metal-coated (i.e., Ti/Au-coated) nanorods were thermally annealed at 573 K for 15 min to ensure ohmic contact of ZnO nanorods. The resulting metal coated ZnO nanorods exhibited much higher *I* values from measurements using the Pulsar indenter tip as shown in Fig. 2, indicating a significant reduction in the contact resistance. Here, although Ti is known to form an Ohmic contact with ZnO^24^, a Schottky barrier was still present as apparent in the non-linear *I-V* curve in Fig. 2, potentially due to the non-ideal surface conditions of incomplete atomic bonds and the conceivable defects or impurities in the nanorods^25^,^26^.

### Compression creep of nanorods

**Figure 3:**
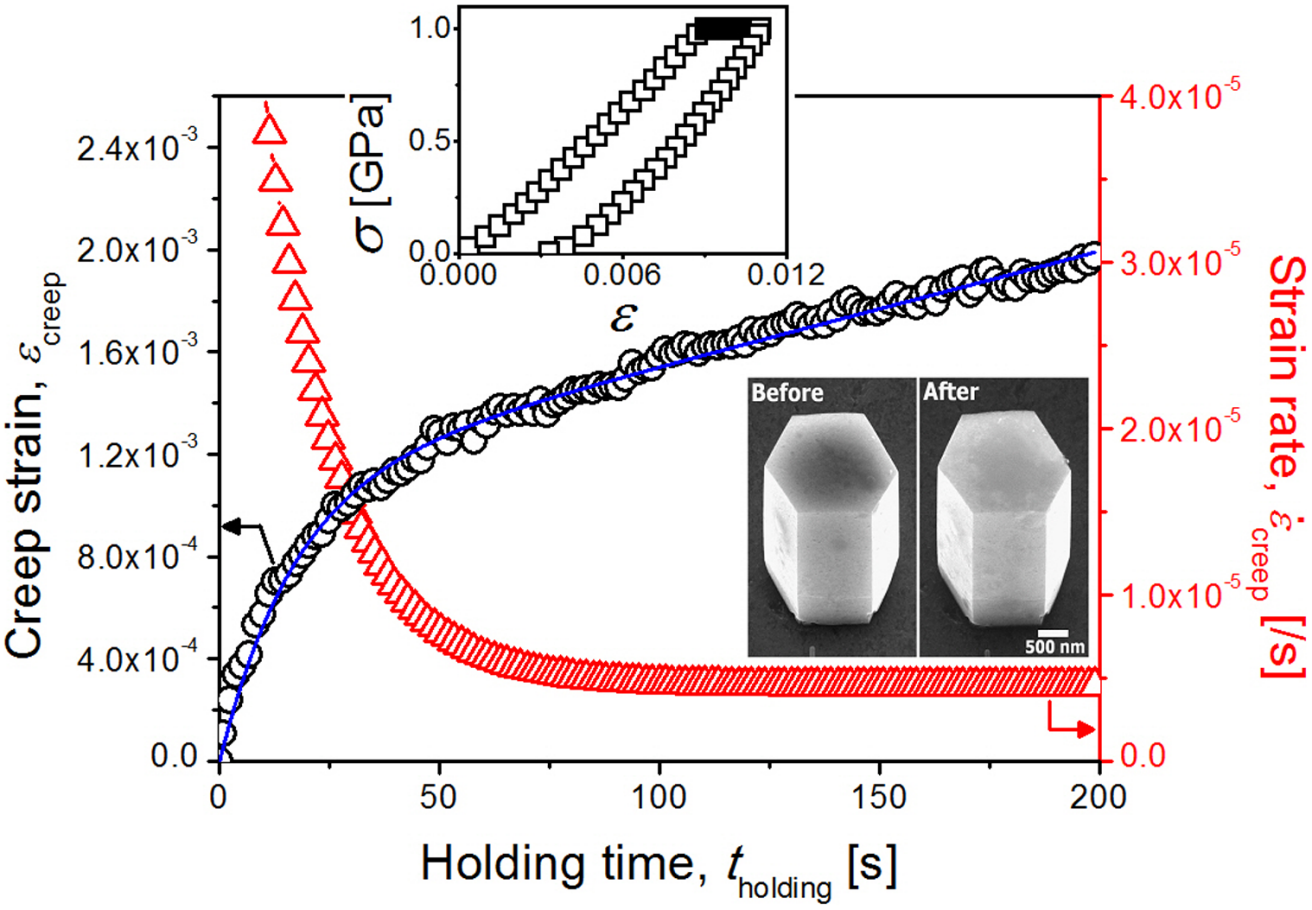
Typical example of *ε*_creep_ (and 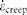) vs. *t*_holding_ curves. Inset shows the representavie *σ*-*ε* curve and the SEM images of a nanorod taken before and after creep tests.

The engineering stress (*σ*) vs. engineering strain (*ε*) of the ZnO nanorods were calculated from the load-displacement (*P*-*h*) data using the simple relations of *σ* ~ *P*/*A*_0_ and *ε* ~ *h*/*l*_0_, where *A*_0_ is the initial top surface area of nanorod and *l*_0_ is the initial height of nanorod. The resulting stress-strain behavior shown in the inset of Fig. 3 indicates an important feature where the creep indeed occurs at ambient temperatures and exhibits a creep strain of up to ~2.0 × 10^−3^ for 200 s of constant stress of 1 GPa, which is still within the elastic regime (i.e., well below the failure strength, ~3.2 GPa^7^). Note that here creep is defined as any type of time-dependent plastic deformation which occurs during load-hold sequence. Thus, the creep strain can be calculated as the increasing amount of strain values during *t*_holding_. The absence of shearing or cracking in the crept rod (in the inset of Fig. 3) indicates that creep deformation was accommodated by the whole body of nanorods.

Representative engineering creep strain (*ε*_creep_) and creep rate (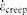) as a function of holding time (*t*_holding_) for ZnO nanorods are shown in Fig. 3. The *ε*_creep_ vs. *t*_holding_ plot is mostly parabolic in nature consisting of two regimes of primary (or transient) and steady-state creep, which is similar to typical high-temperature creep curves of metals and ceramics^27^,^28^. During the primary creep regime, creep strain is relatively high (i.e., large portion of total creep strain is produced in this regime) but decreases with time. In consideration of the approximately linear relation of the thermal-drift-induced displacement and time, the two-regime creep behavior also strongly suggests that the creep is not caused by thermal drift, which is explained in detail in previous creep studies of nanopillars^7^,^29^,^30^. The 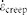 was estimated by first fitting the creep curve according to Garofalo's mathmatical fitting equation developed for uniaxial creep strain, 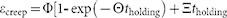, where Φ, Θ, and Ξ are creep constants (for physical meaning of each parameters, see Ref. 28), and then by differentiating the fitted Garofalo's equation with respect to *t*_holding_. The 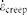 vs. *t*_holding_ plot provided in Fig. 3 suggests the possibility of nanorods approaching close to the steady-state creep condition. Details of the creep deformation and its mechanism is reported in authors' previous study^7^ in which the creep stress exponent (~1) and activation volume (close to ionic volume) were estimated systematically by various experiments including *in-situ*/*ex-situ* creep observations and *in-situ* electric measurements. In the study, we reached the conclusition that the room temperature creep of the nanorods at such low stresses (within elastic regime) may be dominated by the diffusion-controlled mechanisms through the side surface and/or along the interface between the flat punch and the top surface of the rod^7^.

### Creep-induced current change

**Figure 4:**
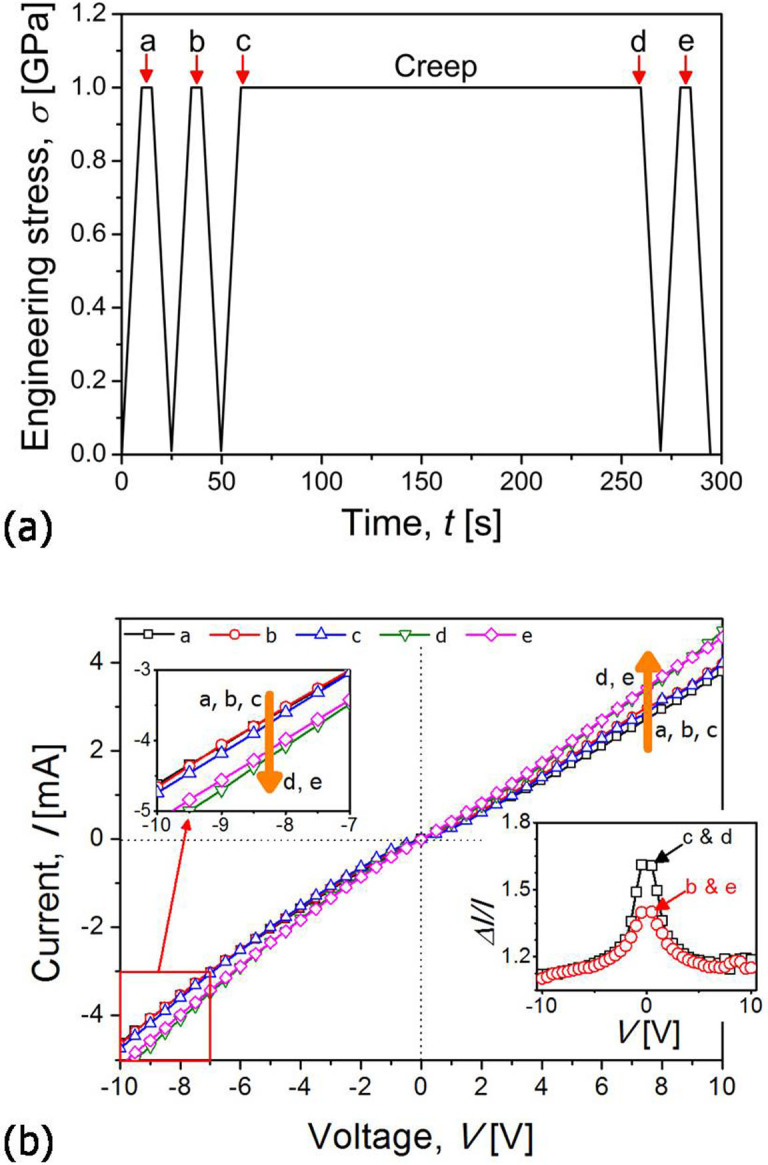
*In-situ* electrical behavior measurement; (a) the loading scheme with indication of the points where *I-V* data were measured; (b) the representative measured *I*-*V* curves with an inset showing the variation in the current change amount as a function of applied voltage.

The changes in *I* of the metal-coated nanorod were measured at five different stages in a test (arrowed “a–e” in Fig. 4a) while varying the applied *V* from −10 V to 10 V to determine the creep-induced current changes. To avoid complications arising from the piezoresistance effect (or the effect of elastic strain on electrical resistance)^31^, all measurement of the change in *I* was performed at a fixed stress of 1 GPa. Figure 4b provides typical example of the *I-V* plots. All three curves obtained before creep tests (“a, b, c”) are repeatable, which indicates that quasi-static loading at the stresses within the elastic regime does not affect current flow. After creep test, the slopes of *I*-*V* curves become higher and the absolute current values are increased in both negative and positive voltage regimes. In order to confirm that the creep strain and the corresponding change in electrical response is permanent, *I*-*V* curves at the point “d” (right after creep) and “e” (at the finish of a test) were compared, which are essentially identical, implying no recovery of the creep-induced changes. The amount of *I* changes, *ΔI/I*, between (1) the onset of creep (“c” in Fig. 4a) and the finish of creep (“d”) and (2) before (“b”) and after (“e”) creep test was summarized in the inset of Fib. 4b. The *ΔI/I* initially decreases with *V* and becomes approximately constant (~1.1–1.15) in the high *V* range (>± 8 V). Asymmetry in the negative and positive *V* regimes is expected to be due to the difference in the Schottky barrier *φ*_B_ between both electrode junctions at the top and bottom of a nanorod.

**Figure 5:**
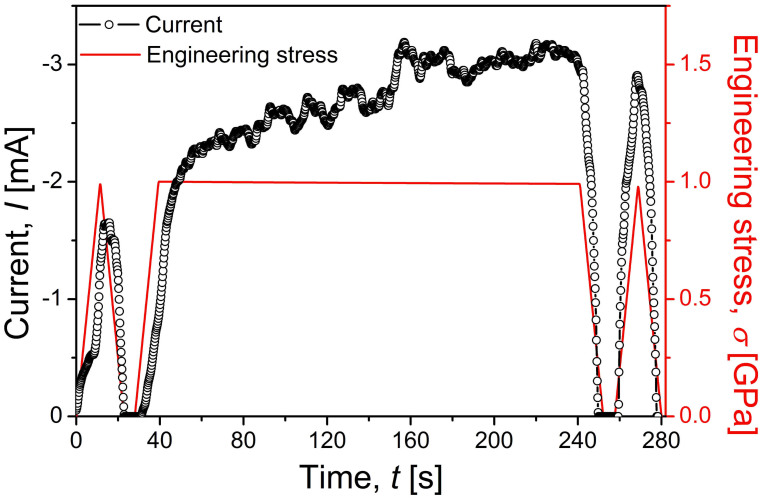
Both *I*-*t* and *σ*-*t* curves obtained during creep tests under constant *V*.

The *I* raised by creep in Fig. 4b could be confirmed by additional *I* measurement test; i.e., *in-situ* *I* measurement at a fixed voltage (−10 V) during creep. As shown in Fig. 5, *I* increases continuously in load-hold sequence, and *I* values re-measured after creep test is higher than that obtained during preloading segment. The *ΔI/I* values were calculated from Fig. 5 as ~0.65 between the before and after creep and ~0.58 between the onset and finish of creep, which are much higher than those estimated from *I-V* relations in Fig. 4b. Although the reason for the fact that *ΔI/I* values are much higher in *in*-*situ* *I* measurement (Fig. 5) than in *I*-*V* measurement (Fig. 4b) is not fully understood yet, it may be partly explained by local temperature increase (or Joule heating effect) due to the exposure of the sample to the high voltage (−10 V) for a relatively long time (~280 s) during the *in*-*situ* test. It was previously shown to be potentially high enough to weld the ZnO nanowire onto electrodes during *in*-*situ* electrical measurement within transmission electron microscopy^32^. In the presence of Joule heating, the *I* may increase with temperature, as suggested by Ip et al.^33^ who reported largely enhanced current with increasing temperature from 303 to 473 K in the case Pt/ZnO Schottky contact. Such a possible temperature change during *in-situ* *I* measurement test can significantly affect thermal stability of the testing. For minimizing the effects of thermal instability and extracting meaningful results, only the results from the *I*-*V* measurements that were taken less than 0.5 s were analyzed. Such a short-time measurement may not induce the serious side effects (such as Joule heating effect), which is supported by the fact that the *I*-*V* data obtained before creep tests (“a, b, c” in Fig. 4b) overlap each other while those taken after creep (“d, e”) do so either.

## Discussion

Current change shown in Fig. 4 and 5 can be explained by microstructure and/or geometry change of nanorods in response to creep deformation. Under the compression creep at room temperature, ZnO nanorods deformed via atomic diffusion along the surface without any evidence for dislocation activities^7^ to result in shorter and wider nanorods. Since change in geometry is directly related with electrical resistance, the creep-enhanced current flow can be quantitatively analyzed by calculating the variation in nanorod resistance *R*_NR_ that arise due to creep deformation. For this purpose, first, one may need to determine a proper electrical transport model because the simple Ohm's law (*R*_NR_ = *I*/*V*) cannot be applied due to the nonlinear *I-V* characteristics. Considering the top (metal and conductive indenter) and the bottom electrode (metal and graphene), the metal-coated ZnO nanorods in this study can be modeled as a metal-semiconductor-metal (M-S-M) structure, in which the electrical transport is governed by “the voltage drop at the reverse-biased Schottky barrier” where electrons flow from M to S^13^,^22^,^34^. In this case, the thermionic-field emission (TFE) and thermionic emission-diffusion (TED) theories are often adopted for analyzing electrical properties of M-S-M structures. In previous publications, both TFE and TED theories have been applied to the undoped ZnO nanomaterial-based M-S-M structures^13^,^32^.

Since it is impossible to identify the nature of the barriers by simply looking at the *I*-*V* curve, selection of proper electrical transport model (i.e., TFE or TED) can be achieved by comparing the experimentally-obtained *I*-*V* curves with the theoretically-developed *I*-*V* relations^13^. According to TFE and TED theory, the *I* through reverse-biased Schottky barrier can be expressed in simplified forms^13^,^22^,^33^:
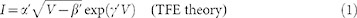
and
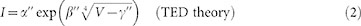
Here *β* (*β*′ and *β*″) and *γ* (*γ*′ and *γ*″) are complex function of Schottky barrier height and temperature, respectively, and can be considered as constants under a given testing condition. Both *α*′ and *α*″ are a function of *A*_EC_ that is the area of electrical contact between M and S at reverse-biased junction. At *V* < 0, the reverse-based junction is at the top surface of nanorod (i.e., between nanorod and flat-ended indenter), *A*_EC_ is the same as the initial contact area of nanorod (*A*_0_). However, at *V* > 0, the junction at the bottom of nanorod (i.e., between graphene and nanorod) becomes reverse-biased, and it is difficult to measure *A*_EC_ between them due to uncertainty of appropriate path for current flow. In this regard, for further analysis given below, we use only the *I*-*V* curves in the negative *V* range, allowing us to apply precise *A*_EC_.

**Figure 6:**
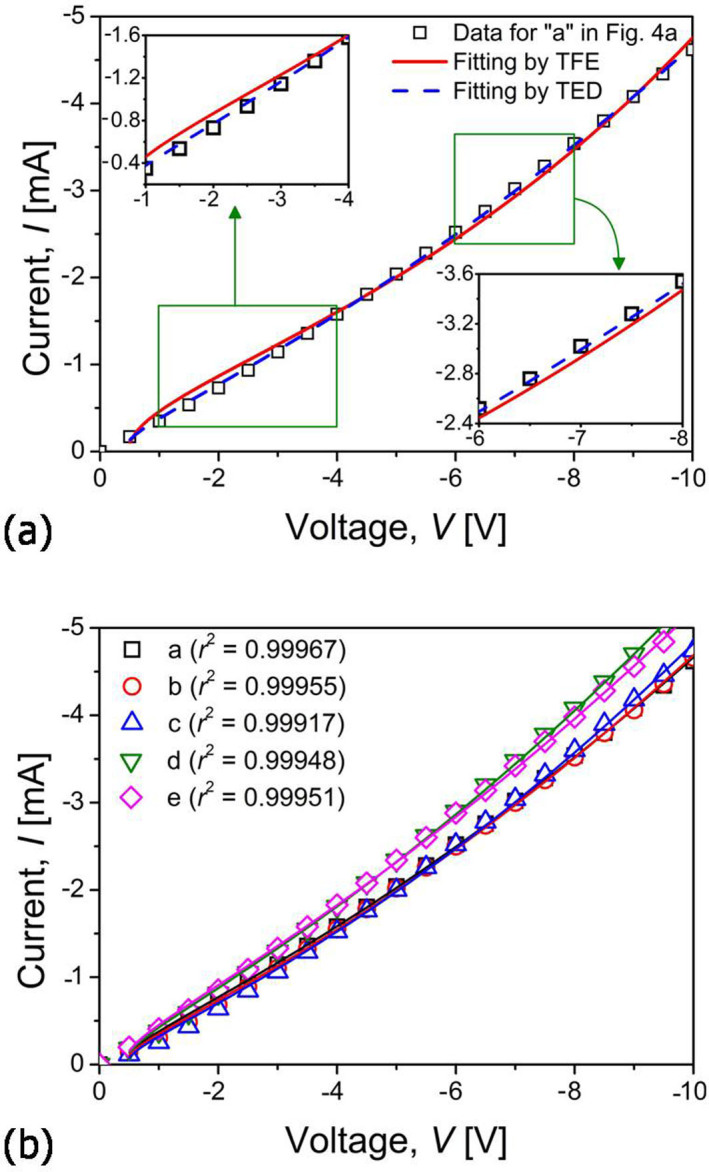
Determination of proper model; (a) example of the *I-V* data fitting by TFE and TED model (for point “a” in Fig. 4a); (b) fitting of each *I*-*V* curve by TED model.

To determine which theory (among TFE and TED) is more appropriate for analyzing the electrical behavior of nanorods, the *I-V* data experimentally measured in the negative *V* range were fitted with Eqs. (1) and (2), respectively. Figure 6a shows an example of the fitting results of the experimental *I-V* data (for “a” point in Fig. 4a). Although both TFE and TED models fit the data reasonably well, TED model gives a better fit to the experimental data; the experimental *I-V* curves of all points (“a–e” in Fig. 4a) are almost perfectly fitted by TED model in Fig. 6b with very high correlation factor (*r*^2^ > 0.99). It was reported for ZnO-based M-S-M structures that, if the measurements are made at room temperature and ZnO has a low doping condition, the predominant transport property at the barrier is TED^13^. Due to the similar testing condition in the present study (such as room temperature testing and undoped nanorods), TED model was selected as a more realistic one applicable to our testing system.

The *R*_NR_ can be estimated from the *I*-*V* curves in consideration of total voltage drop (*V*_T_) which is the same as the applied voltage *V*. Although we assumed *V* ( = *V*_T_) in Eqs. (1) and (2) is equal to *V*_R_ in order to make simpler fitting format, more precisely, the *V*_T_ is the sum of the voltage drops at the reverse-bias barrier (*V*_R_), at the nanorods (*V*_NR_), and at the forward-bias barrier (*V*_F_) in a M-S-M structure; i.e., *V*_T_ = *V* = *V*_R_ + *V*_NR_ + *V*_F_. While *V*_F_/*V*_T_ is almost always negligible, *V*_NR_/*V*_T_ increases with increasing *V* and becomes predominant at high *V* regime^32^,^34^.

**Figure 7:**
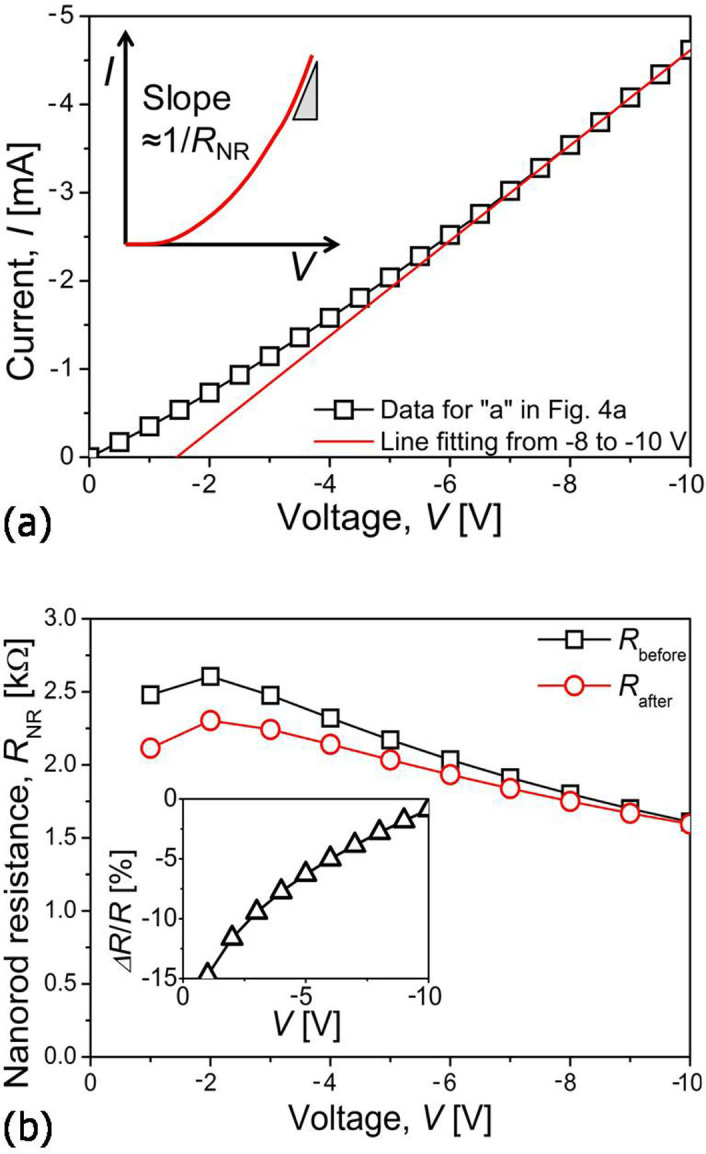
Calculation of creep-induced resistance change; (a) fitting of the *I-V* data in high *V* regime (for point “a”), of which slope is a reciprocal *R*_NR_ as schematically shown in the inset. (b) *R*_NR_ vs. *V* plot with an inset showing the relation of *ΔR*/*R*_NR_ vs. *V*.

In the present study, we adopted two methods for estimating the *R*_NR_. The first method is for high *V* regime in which (1) *V* ≈ *V*_NR_ and (2) the *I*-*V* relation becomes more linear. Thus, following Ohm's law, the slope of the *I*-*V* curves in this regime (i.e., d*I*/d*V* ≈ d*I*/d*V*_NR_) is ~1/*R*_NR_, as illustrated in the inset of Fig. 7a. A representative example of the application of this method (for point “a” in Fig. 4a) is provided in Fig. 7a. The experimental data in high *V* range (−8 to −10 V) shows a good linear fitting, suggesting that the application of this method may be valid. From Eq. (2), *R*_NR_ can be quantified as:
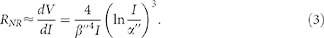
The values of *α*″ and *β*″ can be determined by fitting the experimental *I*-*V* data with Eq. (2).

Figure 7b exhibits the variations in the estimated *R*_NR_ values before creep (point “b”), *R*_before_, and after creep (point “e”), *R*_after_, as a function of negative voltage. Except for the low voltage regime data, both *R*_before_ and *R*_after_ decrease simultaneously with *V*. The ratio of *ΔR*/*R*_NR_ (where *ΔR* = *R*_after_ − *R*_before_) is provided in the inset of Fig. 7b. Since this first method is valid in high *V* regime (where *V* ≈ *V*_NR_), *ΔR*/*R*_NR_ was calculated at −10 V which is the highest applied *V* in this study. Resultantly, *R*_NR_ was found to decrease from ~1.609 to ~1.595 kΩ by creep deformation and thus *ΔR*/*R*_NR_ was about −0.89%.

The second method we applied here is not necessarily limited to high voltage regime (where *V* ≈ *V*_NR_) and is based on more general relation *V* ≈ *V*_R_ + *V*_NR_^35^. If *V* in Eq. (2) is replaced by (*V* − *V*_NR_), and by the procedure similar to that used for derivation of Eq. (2), one can get an equation of *V* as a function of *R*_NR_:
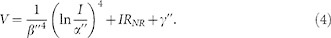
By fitting the *I*-*V* data to this equation, the *R*_NR_ can be directly determined. The *R*_NR_ before and after creep tests was calculated as 1.0958 and 1.0883 kΩ, respectively. Accordingly, the *ΔR*/*R*_NR_ is about −0.68%, which is very close to the value obtained by first method. In order to check how much the *ΔR*/*R*_NR_ varies with sample, the ratios of other nanorods were additionally measured. Interestingly, although the absolute resistance value was different for each nanorod, the obtained *ΔR*/*R*_NR_ values were very similar; ~ −1.32 ± 0.67% and ~ −1.08 ± 0.8% for the first and second method, respectively. This similarity may result from the similar creep condition and creep strain amount.

During the deformation under compressive loading, the height of nanorod (*l*) decreases and both the equivalent diameter (*d*) and cross-sectional area (*A*) increase, which results in reduction in *R*_NR_ according to
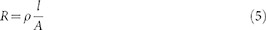
where *ρ* is the resistivity. Based on this, we develop a simple analytical model to predict the creep-induced *ΔR*/*R*_NR_ from the engineering creep strain *ε*_creep_ ( = *h*_creep_/*l*_0_ where *h*_creep_ is the creep displacement). For the sake of simplicity, here we assumed that, upon compressive creep, a geometry change can occur uniformly in whole nanorod body through diffusion-based mechanism^7^ without accompanying any change in defect density and electrical resistivity.

There are two different types of deformation during creep test; elastic deformation in the loading sequence and permanent deformation in the load-hold sequence. The *l* at the end of creep (point “d” in Fig. 4a) can be given as
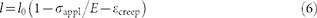
where *σ*_appl_ is the applied stress (1 GPa in this study), and *E* is elastic modulus (~124 GPa for ZnO^36^). The radial elastic strain ( = Δ*d*/*d*_0_ where Δ*d* is the amount of diagonal length change) can be given as (−*ν*) times the axial elastic strain ( = *σ*_appl_/E) where *ν* is the Poisson's ratio (0.356 for ZnO^37^). In the case of creep (or permanent) deformation, based on strong possibility of volume conservation, we roughly assumed Δ*d*/*d*_0_ = 0.5(*σ*_appl_/E). Similar to Eq. (6), the *d* at the end of creep can be expressed as
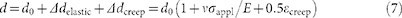
where Δ*d*_elastic_ and Δ*d*_creep_ are the amount of *d* change by elastic and creep deformation, respectively. On the other hand, the cross-sectional area of a nanorod can be calculated as
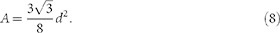


Finally, the *R*_NR_ of crept nanorods (i.e., *R*_after_) can be obtained as a function of *ε*_creep_ by putting Eqs. (6)–(8)Eqs. (6)–(8)Eqs. (6)–(8) into Eq. (5):
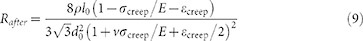
from which the *R*_NR_ before creep test (*R*_before_) can be calculated with *ε*_creep_ = 0. An unknown value in the equation is *ρ* which may not vary by diffusion creep^7^. To take *ρ* out of the evaluating expression, it is better to calculate the ratio of *R*_after_/*R*_before_ rather than just *R*_after_:
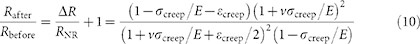
By putting *ε*_creep_ (obtained at Fig. 3 as ~2.0 × 10^−3^) into Eq. (10), predicted *ΔR*/*R*_NR_ by creep is −0.4% which is reasonably close to the values obtained from experiments in previous section (−0.89 and −0.68% depending on the method). Small difference between the predicted values and the experimental values may come from the uncertainty in the full mechanical contact possibly due to the surface roughness at the top surface of the nanorod^38^.

Semiconducting ceramic materials (such as ZnO, examined in the present study) usually have extremely low dislocation mobility in the elastic stress regime at room temperature. However, the outcomes of this study have demonstrated that significant creep deformation can occur through diffusional atomic movement along the surface, especially when the sample dimension is reduced down to the nanoscale. As a result of pronounced creep deformation, the electrical response of the nanomaterials can fluctuate, which needs to be carefully considered when applying the nanostructures to different devices having small tolerance in resistance fluctuations. It should be noted that the prediction for the nanorod resistance is subject to fluctuations that may arise due to different degrees of creep being induced by different loading or structural conditions. For example, more variance in electrical properties is expected at higher stresses, longer loading time, and smaller nanomaterial size since the creep deformation becomes more pronounced under these conditions^7^. In this regard, the model suggested here is expected to be useful for predicting the creep-induced resistance change of nanomaterials that can help in designing and manufacturing of nanomaterial based devices with long-term reliability and stability.

In this study, we have systematically explored the change in electrical properties during nanoscale compression creep deformation. *In*-*situ* mechanical-electrical coupled tests were performed on ZnO nanorods at the low stress within elastic regime. It was revealed that the creep deformation increases the current due to increase in the cross sectional area as a result of diffusion mechanisms at room temperature aided by surface diffusion. Based on the thermionic emission-diffusion (TED) theory of the M-S-M structure, the creep-induced resistance change of nanorod (*ΔR*/*R*_NR_) was calculated to be −0.89% and −0.68% using two different methods of taking *V*_T_ to be either the same as *V*_NR_ or the summation of *V*_R_ + *V*_NR_, respectively. Finally, we proposed a simple analytical model to predict the *ΔR*/*R*_NR_ as a function of creep strain by considering the change in the nanorod geometry through diffusion creep, and the predictions were in an excellent agreement with the experimentally determined values.

## Methods

**Figure 8:**
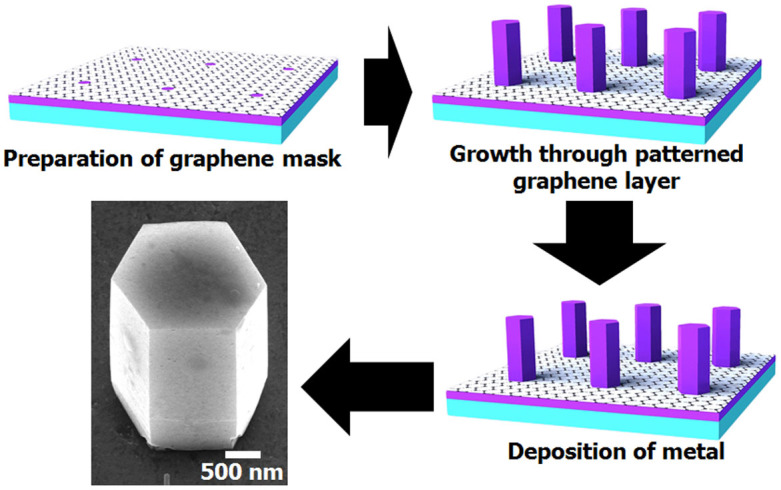
Schematic illustration showing the preparation procedures for testing structures including SEM images of metal-deposited ZnO nanorods.

Single crystal ZnO nanorods were synthesized through hydrothermal method using epitaxial ZnO seed layer prepared by pulsed laser deposition onto (0001) Al_2_O_3_. For controlling size and position of nanorods, growth mask of photoresist (PR) AZ1512 layer patterned by photo-lithography was used. During bottom-up growth with the presence of a mask, however, lateral overgrowth typically occurs above the patterned holes, which can result in necked region at the bottom of the nanorod. To minimize the formation of the necked region in the ZnO nanorod, graphene layer was used as a mask instead of a thick photoresist layer. Graphene layer was transferred onto the seed layer followed by PR coating. A pattern consisting of an array of holes was defined in graphene through the sequence of photolithography, etching of the exposed graphene with O_2_ plasma, and removal of PR layer with acetone. Hydrothermal growth was carried out in an aqueous solution containing 0.025 M zinc nitrate hexahydrate [Zn(NO_3_)_2_·6H_2_O, Sigma-Aldrich] and 0.025 M hexamethylenetetramine (C_6_H_12_N_4_, Sigma-Aldrich) at 70°C for ~12 h. More detailed process is described in our previous study^7^. The resulting nanorods are hexagonal in cross section with initial equivalent diameter (or diagonal length of the hexagon), *d*_0_, of ~2000 nm and aspect ratio of ~2:1–3:1. Additionally, ~10 nm-thick Ti/Au is uniformly deposited on the top surface of whole samples following the annealing at 573 K for 15 min in N_2_ atmosphere to improve electrical conductivity of the contact surfaces. Schematic illustration of the nanorod synthesis procedure and the typical shape of an as-grown nanorod are shown in Fig. 8.

*In-situ* electro-mechanical coupled tests were performed by using TI-750 Ubi nanoindenter (Hysitron Inc., Minneapolis, MN) equipped with NanoECR system for applying and measuring electrical signals (*V* and *I*). Two different flat-ended tips including a commonly used boron-doped diamond tip and a metal-carbide indenter called the “Pulsar” (Hysitron Inc., Minneapolis, MN) were used, although the main observation was conducted on the results with Pulsar tip. Specimens were attached to copper holders with silver paste. The schematic of the testing setup is provided in Fig. 1 where the direction of current (and electron) flow is indicated.

During the creep tests, load was held at maximum load, *P*_max_, for 200 s and loading/unloading time was set to 10 s. The applied *P*_max_ was determined to produce a stress of 1 GPa which is far below the failure strength of ZnO nanorods (~3.2 GPa for ~2000 nm-diameter nanorods^7^). The *I*-*V* characteristics were measured at *P*_max_ in the voltage range from −10 to 10 V. Each measurement took only ~0.47 s which was thought to be short enough for being free from thermal drift in the system; because creep tests were performed at a thermal drift rate lower than 0.1 nm/s, the drift-induced displacement during *I*-*V* measurement for ~0.47 s must be less than ~0.047 nm which is almost negligible. In addition, creep tests were also conducted under a fixed *V* (of −10 V) to *in-situ* record the *I* change. Before and after creep tests, morphology of nanorods were examined using a scanning electron microscopy (SEM), JSM-6330F (JEOL Ltd., Tokyo, Japan).

## Author Contributions

Y.-J. carried out all experiments presented in this manuscript with a help of I.-C. for creep tests, T.G. for in-situ electrical measurement, and S.W. and W.I. for specimen preparation. J.-i. and S.M. supervised the project. All authors contributed to and discussed the manuscript.

## Additional Information

**How to cite this article**: Kim, Y.-J. *et al.* Time-dependent mechanical-electrical coupled behavior in single crystal ZnO nanorods. *Sci. Rep.* 5, 9716; DOI:10.1038/srep09716 (2015).

## Supplementary Material

Supplementary Information Supplementary information
