# Supplementary material for: Time-dependent mechanical-electrical coupled behavior in single crystal ZnO nanorods
Source: Sci Rep. 2015 May 18;5:9716. doi: 10.1038/srep09716 (PMC4434914; doi:10.1038/srep09716)
Supplement: Supplementary Information [file srep09716-s1.doc]

*Supplementary information for*

**Time-dependent mechanical-electrical coupled behavior in single crystal ZnO nanorods**

Yong-Jae Kim,^1^ Tae Gwang Yun,^2^ In-Chul Choi,^1^ Sungwoong Kim,^1^ Won Il Park,^1^

Seung Min Han,^2^,* Jae-il Jang,^1^,*

^1^Division of Materials Science and Engineering, Hanyang University, Seoul 133-791, Korea

^2^Graduate School of EEWS, Korea Advanced Institute of Science and Technology, Daejeon 305-701, Korea

* Corresponding authors: jijang@hanyang.ac.kr (J.-i. Jang), smhan01@kaist.ac.kr (S. M. Han)

Since it is possible that top and bottom of nanorods can be short-circuited by deposited metal layer on the side surface of nanorods, EDS analysis were performed for the ZnO nanorod at the location marked in Fig. S1a. As shown in Fig. S1b, metal components of Au and Ti were found only on the top surface of nanorod, while any metals were detected on its side surface (see Fig. S1c).


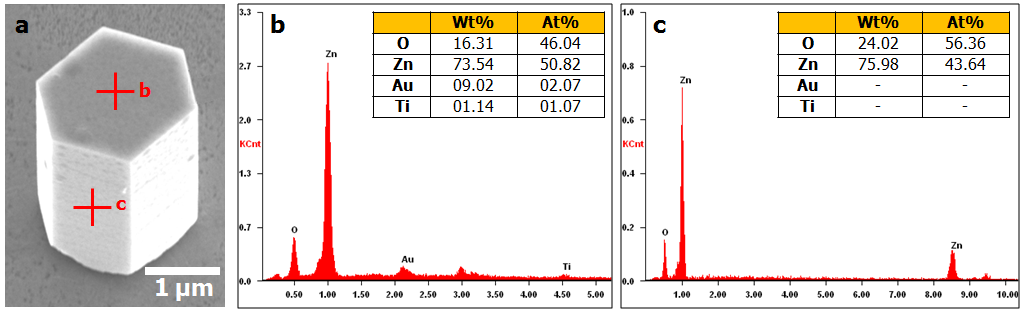


Fig. S1. (a) SEM image of metal-deposited ZnO nanorods. EDS results obtained from (b) top surface of nanorod and (c) its side surface.

In addition, the resistivity (*ρ* = *RA*/*l*) of the nanorod is calculated to be ~ 1.2×10^-3^ and 0.8×10^-3^ Ω·m from method 1 and 2 as shown in Tabe S1, which is again comparable with the values in the literatures of ~10^-4^-10^-1^ Ω·m for ZnO nanomaterials [S1].

Table S1. Calculation of ZnO nanorod resistivity

|  | ***R*_NR_ [kΩ]** | ***d* [μm]** | ***l* [μm]** | ***ρ* [Ω·m]** |
| --- | --- | --- | --- | --- |
| **Method 1** | 1.609 | 2.1 | 3.7 | 1.246×10^-3^ |
| **Method 2** | 1.0958 |  |  | 0.848×10^-3^ |

Based on the evidences described above and included in the manuscript, the electrical responses we measured is resulted from the ZnO nanorods rather than short-circuited Au layer on the surface of nanorod.

**References**

[S1] J. He et al., *J. Am. Chem. Soc.*, 127 (2005) 16376.
